# A polymorphism in the interleukin-4 receptor affects the ability of interleukin-4 to regulate Th17 cells: a possible immunoregulatory mechanism for genetic control of the severity of rheumatoid arthritis

**DOI:** 10.1186/ar3239

**Published:** 2011-02-04

**Authors:** Susan K Wallis, Laura A Cooney, Judith L Endres, Min Jie Lee, Jennifer Ryu, Emily C Somers, David A Fox

**Affiliations:** 1 Division of Rheumatology and Rheumatic Diseases Research Core Center, Department of Internal Medicine, University of Michigan, 1500 East Medical Center Drive, Ann Arbor, MI 48109, USA

## Abstract

**Introduction:**

Rheumatoid arthritis (RA) is now suspected to be driven by pathogenic Th17 cells that secrete interleukin (IL)-17 and can be regulated by IL-4. A single-nucleotide polymorphism (SNP), I50V, in the coding region of the human IL-4 receptor (IL-4R) is associated with rapid development of erosive disease in RA. The present study was undertaken to determine whether this SNP renders the IL-4R less able to transduce signals that regulate IL-17 production.

**Methods:**

Peripheral blood mononuclear cells were activated under Th17-stimulating conditions in the presence or absence of IL-4, and IL-17 production was measured by both enzyme-linked immunosorbent assay (ELISA) and flow cytometry. Serum IL-17 was also measured by ELISA. Paired comparisons were performed using the two-tailed *t*-test. IL-4 receptor gene alleles were determined by polymerase chain reaction.

**Results:**

In healthy individuals, IL-4 significantly inhibited IL-17 production by cells from subjects with the I/I genotype (*P *= 0.0079) and the I/V genotype (*P *= 0.013), but not the V/V genotype (*P *> 0.05). In a cross-sectional sample of patients with established RA, the magnitude of the *in vitro *effect of IL-4 was lower and was not associated with a specific IL-4R allele. Serum IL-17 levels were higher in RA patients than in healthy individuals, as was the percentage of CD4^+ ^cells that produced IL-17.

**Conclusions:**

These results indicate that an inherited polymorphism of the *IL-4R *controls the ability of the human immune system to regulate the magnitude of IL-17 production. However, in established RA, this pattern may be altered, possibly due to secondary effects of both RA itself as well as immunomodulatory medications. Ineffective control of Th17 immune responses is a potential mechanism to explain why IL-4R is an important severity gene in RA, but this issue will require careful study of a cohort of new-onset RA patients.

## Introduction

Until recently, CD4^+ ^lymphocytes were thought to contain two distinct lineages of effector cells, the Th1 and Th2 subsets that are defined by secretion of either interferon (IFN)-γ or interleukin (IL)-4. This paradigm has been modified to now include a third CD4^+ ^T-cell population, the Th17 cells [1,2]. Th17 cells are critical for autoimmune inflammation in a variety of murine models of human disease, such as experimental autoimmune encephalomyelitis (EAE) and collagen-induced arthritis (CIA) [3-5].

Unique mechanisms control the development of these cells. The cytokines IL-6 and tumor growth factor (TGF)-β are crucial for the generation of Th17 cells in the mouse [6-8], while IL-1β, IL-6 and IL-23 induce and maintain the differentiation of human Th17 cells [9,10]. Accumulating evidence suggests that Th17 cells play a central role in the development of human autoimmune diseases, including RA, inflammatory bowel disease and multiple sclerosis [11].

Th17 cell development and cytokine secretion are downregulated *in vitro *by IFN-γ and IL-4 produced by Th1 and Th2 cells, respectively [1,2,12,13]. Understanding the mechanisms of Th17 regulation in human disease is essential for the development of novel, targeted therapies and to guide therapeutic decision-making.

Several findings suggest that the Th2 cytokine IL-4 and its receptor may be of particular interest in the control of Th17-induced inflammation. In mice, the genetic absence of IL-4 leads to more severe arthritis in the CIA model [14]. Conversely, dendritic cells transfected with a retroviral vector that drives expression of IL-4 reduced the severity of CIA and suppressed IL-17 production in secondary responses to type II collagen [15,16]. Suppression of IL-17 production by type II collagen-specific T cells was seen early in CIA, but T cells from established late CIA were refractory to inhibition of IL-17 production by IL-4 [16]. Exosomes derived from IL-4-expressing dendritic cells were also found to be therapeutic in CIA [17].

In humans, a diminished response to IL-4 is thought to contribute to autoimmune inflammation [18]. A single-nucleotide polymorphism (SNP) in the coding region of the *IL-4R *governs the presence of isoleucine (I) versus valine (V) at position 50 in the amino acid sequence. This polymorphism in *IL-4R *is functionally important because it affects the strength of signaling through the receptor [19,20].

Additional evidence for a crucial role of IL-4 in regulating human RA comes from a report of the effect of IL-4 receptor gene (*IL-4R*) polymorphisms on the course and severity of RA. Prots *et al*. [21] studied the role of two *IL-4R *SNPs in RA susceptibility and severity in a cohort of controls and RA patients with erosive disease. In their study, each polymorphism was in Hardy-Weinberg equilibrium, and *IL-4R *was not found to be an RA susceptibility gene. The I50 and V50 alleles were in an approximately 1:1 ratio in both the RA and control groups. Two years after the onset of disease 68% of RA patients homozygous for the V50 allele had radiographically visible bone erosion compared to 37% of the patients homozygous for the I50 allele. Heterozygotes had an intermediate level of radiographic severity. The V50 homozygous patients demonstrated weaker signaling through the IL-4R as measured by *GATA-3 *transcription and IL-12R expression in cultured T cells [21]. A second polymorphism, located elsewhere in *IL-4R*, did not control RA severity. These findings suggest that a unique *IL-4R *polymorphism may predict disease outcome in RA. Since tight control of the clinical activity of RA substantially improves patient outcomes [22,23], identification of patients who require early aggressive treatment by genotyping for severity has the potential to enhance patient care.

On the basis of these considerations, we hypothesized that a hypofunctional IL-4R would allow unchecked Th17 differentiation and Th17-driven inflammation. We sought to show that Th17 cells derived from healthy V50 homozygotes would be less susceptible to suppression of IL-17 production by IL-4 compared to I50 homozygotes or heterozygotes. We also undertook a pilot cross-sectional study of patients with established RA to assess the relationship between *IL-4R *genotype, disease activity and regulation of IL-17 production *in vivo *and *in vitro*. Our data indicate that deficiency in regulation of IL-17 production is a possible mechanism to explain the association of an *IL-4R *polymorphism with RA severity.

## Materials and methods

### Study populations and clinical evaluation

Twenty patients with established RA and 26 healthy individuals were enrolled in the study. The average age of the healthy individuals was 40.6 years (range, 21 to 62 years), and this group included 12 females and 14 males. The characteristics of the RA patients are summarized in Table S1 (Additional file 1). Health assessment questionnaires were completed by each patient, and disease activity scores were calculated on the basis of a 28-joint count and a visual analogue scale. Thirty milliliters of blood were collected from each subject. Twenty milliliters were saved for cell culture, 5 ml were saved for DNA isolation and genotyping and 5 ml were saved for serum. All study participants provided written informed consent. The research protocol was approved by the University of Michigan Institutional Review Board.

### DNA isolation and genotyping

DNA was isolated from peripheral blood cells using the Qiagen QIAmp Blood Midi kit (Qiagen, Chatsworth, CA, USA) by a spin protocol according to manufacturer's instructions. Genotypes for I50V SNP of the *IL-4R *were determined by allele-specific real-time polymerase chain reaction (RT-PCR) using TaqMan Genotyping Assays (Applied Biosystems, division of Life Technologies, Carlsbad, CA, USA). The National Center for Biotechnology Information SNP reference for the I50V allele is rs1805010, and the nucleotide sequence surrounding the probe is CTGTGTCTGCAGAGCCCACACG TGT[A/G]TCCCTGAG AACAACGGAGGCGCGGG. RT-PCR was performed for allelic discrimination using a quantitative fluorescence measurement system.

### Cell culture

Peripheral blood mononuclear cells (PBMCs) were isolated from heparinized peripheral whole blood of RA patients and healthy controls by gradient centrifugation over Histopaque-1077 (Sigma, St. Louis, MO, USA). Cell cultures were performed in RPMI 1066 medium (Lonza, Basel, Switzerland) with 10% fetal bovine serum, 1% penicillin G/1% streptomycin and 2% L-glutamine. PBMCs were activated with Orthoclone OKT3 (anti-CD3, produced in the University of Michigan Hybridoma Core) 1 μg/ml and either Th17-stimulating conditions alone (IL-23, 10 ng/ml; IL-1β, 5 ng/ml; IL-6, 10 ng/ml) or Th17-stimulating conditions with the addition of IL-4 (50 ng/ml). Cells were left in culture for 96 hours. Supernatants were collected from each culture condition and stored at -80°C for analysis by ELISA.

### Surface and intracellular staining

On day 5 of culture, the cells were restimulated with phorbol myristate acetate (5 ng/ml) and ionomycin (500 ng/ml) for 1 hour prior to addition of brefeldin A (10 μg/ml) for 5 more hours. The cells were washed and 1 × 10^6 ^cells per sample were used for staining. Cells were first blocked with 20 μl of 10% human serum/10% mouse serum in PBS at 4°C for 15 minutes. The cells were surface-stained with antigen-presenting cell (APC)-labeled mouse anti-human CD4 (BD Bioscience (Palo Alto, CA, USA) or APC-conjugated mouse immunoglobulin G_1 _(mIgG_1_) isotype control (Ebioscience, San Diego, CA, USA), at 4°C for 30 minutes, washed twice with cold 2% newborn calf serum/phosphate-buffered saline (NCS/PBS) buffer and fixed overnight in 4% paraformaldehyde. The cells were then permeabilized with 0.5% saponin in 2% NCS/PBS. Intracellular cytokine staining was performed using fluorescein isothiocyanate (FITC)-labeled anti-human IFN-γ (BD Bioscience) and phycoerythrin (PE)-labeled anti-human IL-17A (Ebioscience), or FITC-conjugated mIgG_1 _isotype control (Ebioscience) and PE-conjugated mouse IgG_1 _isotype control (Ancell, Bayport, MN, USA). Samples were run on a BD Biosciences FACS Calibur flow cytometer and analyzed by CellQuest Pro (BD Bioscience).

### ELISA

Both culture supernatants and fresh sera were analyzed by ELISA for IL-17A levels. Flat-bottomed, high binding, 96-well plates (Corning Costar, Lowell, MA, USA) were coated overnight at 4°C with anti-human IL-17-purified antibody (Ebioscience) diluted to 1:500 with 0.1 M carbonate buffer, pH 9.4. On day 2, the plates were washed three times with 1 × PBS/0.05% Tween at 200 μl per well and blocked using 200 μl of PBS with 10% fetal calf serum per well for 2 hours. The plates were then washed three times with 200 μl of 1 × PBS/0.05% Tween per well. The standard curve was created in duplicate starting with a concentration of 2,000 pg/ml and serial twofold dilutions to 7.8 pg/ml. Supernatants and sera were assayed in triplicate at 100 μl per well, both undiluted and at a 1:5 dilution. The samples were then refrigerated at 4°C overnight, after which they were washed five times with 200 μl of 1 × PBS/0.05% Tween per well. A secondary biotinylated anti-IL-17 antibody and the detection reagent streptavidin horseradish peroxidase were added to each well and incubated at room temperature for 2 hours. The plates were washed seven times with 1 × PBS/0.05% Tween with 1-minute soaks between washes. Tetramethylbenzidine 100 μl were added to each well, and plates were kept in the dark at room temperature for 10 to 30 minutes. Stop solution, 2 N H_2_SO_4_, was added to each well. ELISA plates were read by a Synergy HT plate reader (Biotek, Winsooki, VT, USA) and analyzed by KC4 software (Biotek).

### Statistical analysis

The data were analyzed with GraphPad Prism version 4.02 software (GraphPad Software Inc., San Diego, CA, USA). Paired comparisons were performed using a two-tailed *t*-test. Values of *P *≤ 0.05 were considered significant. Dot plots were generated in CellQuest Pro.

## Results

### IL-17 production in culture supernatants

**Figure 1:**
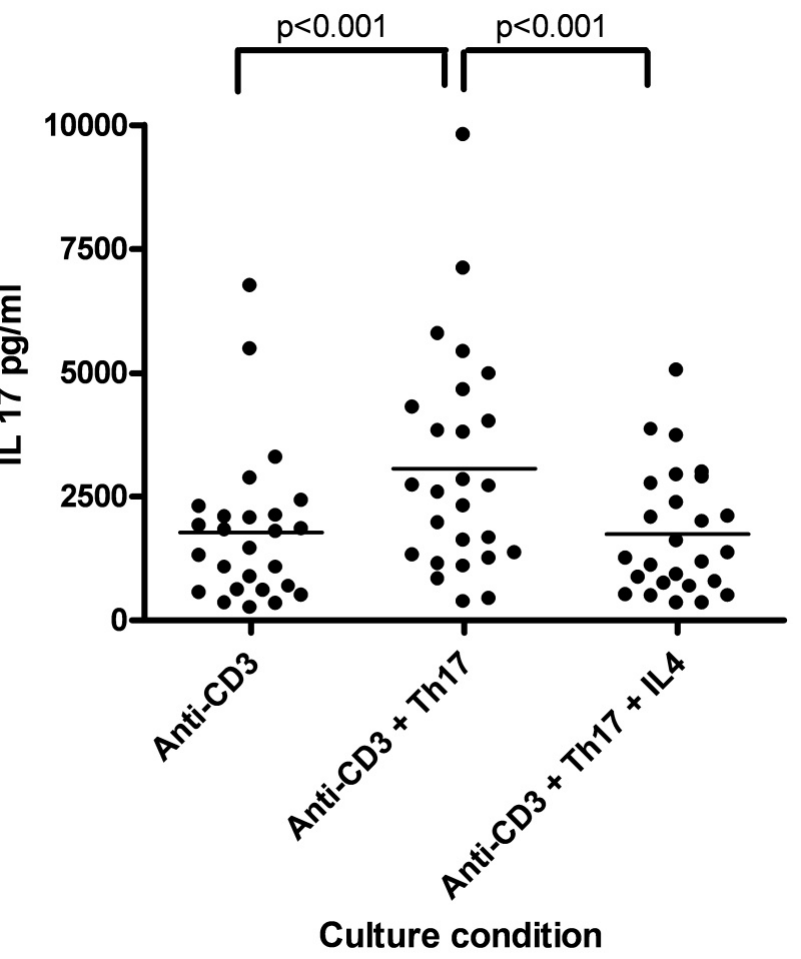
**Regulation of interleukin (IL)-17 production *in vitro***. IL-17A levels (pg/ml) measured by enzyme-linked immunosorbent assay (ELISA) from supernatants taken from three different culture conditions in healthy individuals. Calculated *P *values are from two-tailed *t*-tests between IL-17 levels measured by ELISA from cultures containing anti-CD3, anti-CD3 plus Th17 stimulatory conditions and anti-CD3 plus Th17 stimulatory conditions with the addition of IL-4.

We measured IL-17 secretion by ELISA of lymphocyte culture supernatants. In the healthy individuals there was a significant increase in the IL-17 level after the addition of Th17-stimulatory cytokines over baseline T cell stimulation with anti-CD3 (*P *< 0.01), and there was a significant decrease in the measured IL-17 level with the addition of IL-4 to the Th17-stimulatory conditions (Figure 1).

**Figure 2:**
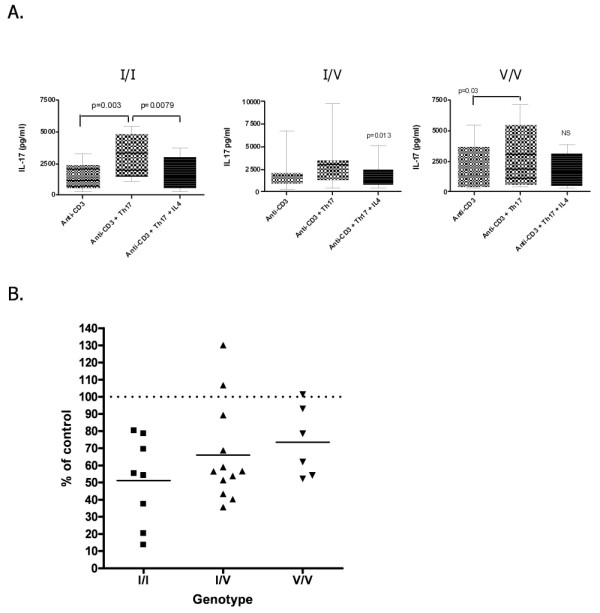
**Inhibition of interleukin (IL)-17 production by IL-4: effect of *IL-4R *genotype**. **(A) **Healthy control group IL-17 measured by enzyme-linked immunosorbent assay (ELISA) of culture supernatants. Comparison of Th17 conditions with or without IL-4: I/I, *P *= 0.0079; I/V, *P *= 0.0013; V/V, *P *= NS. Paired comparisons were performed using a two-tailed *t*-test. **(B) **Proportion of IL-17 inhibition by IL-4. Assuming 100% to be the maximal IL-17 production (measured by ELISA) in supernatants of cultures containing anti-CD3 and Th17 stimulatory conditions, the percentage change from baseline after the addition of IL-4 to cultures of peripheral blood mononuclear cells is shown. I, isoleucine; V, valine.

We then further examined these groups by specific genotype. In the I/I genotype group, addition of IL-4 led to a significant reduction in IL-17 production by cells that had been stimulated under Th17 conditions (*P *< 0.01). There was also a significant reduction in IL-17 production after the addition of IL-4 to cells from the I/V genotype group (*P *< 0.05). However, IL-4 was unable to significantly reduce IL-17 production in cell cultures from the V/V genotype group when the data were analyzed using paired comparisons (Figure 2A and 2B).

### Cross-sectional pilot study of RA patients

Of the 20 RA patients (85% women and 15% men), 4 were homozygous for isoleucine, 6 were heterozygous and 10 were homozygous for valine at amino acid 50 of the IL-4R (Table S1 in Additional file 1). The mean disease activity score (DAS) for the patients with an I/I genotype was 3.1, representing low disease activity. The mean DAS for the patients with the I/V genotype was 3.9, or moderate disease activity, and for the patients with the V/V genotype the mean DAS was 4.2, or high to moderate disease activity. The differences between these groups were not statistically significant, but suggest a trend toward association of the V allele with more active disease, notwithstanding the aggressive treatment that these patients were receiving.

There was not a significant increase in IL-17 production in RA patients in Th17-skewing conditions versus culture with anti-CD3 alone (*P *= 0.13) (Figure S1 in Additional file 1). IL-4 did suppress IL-17 production *in vitro*, albeit not to the extent seen in healthy controls. Comparing the RA groups, the extent of suppression of IL-17 production by IL-4 was intermediate and appeared to be similar among all genotype groups (Figure S2 in Additional file 1).

### Enumeration of Th17+ cells

**Figure 3:**
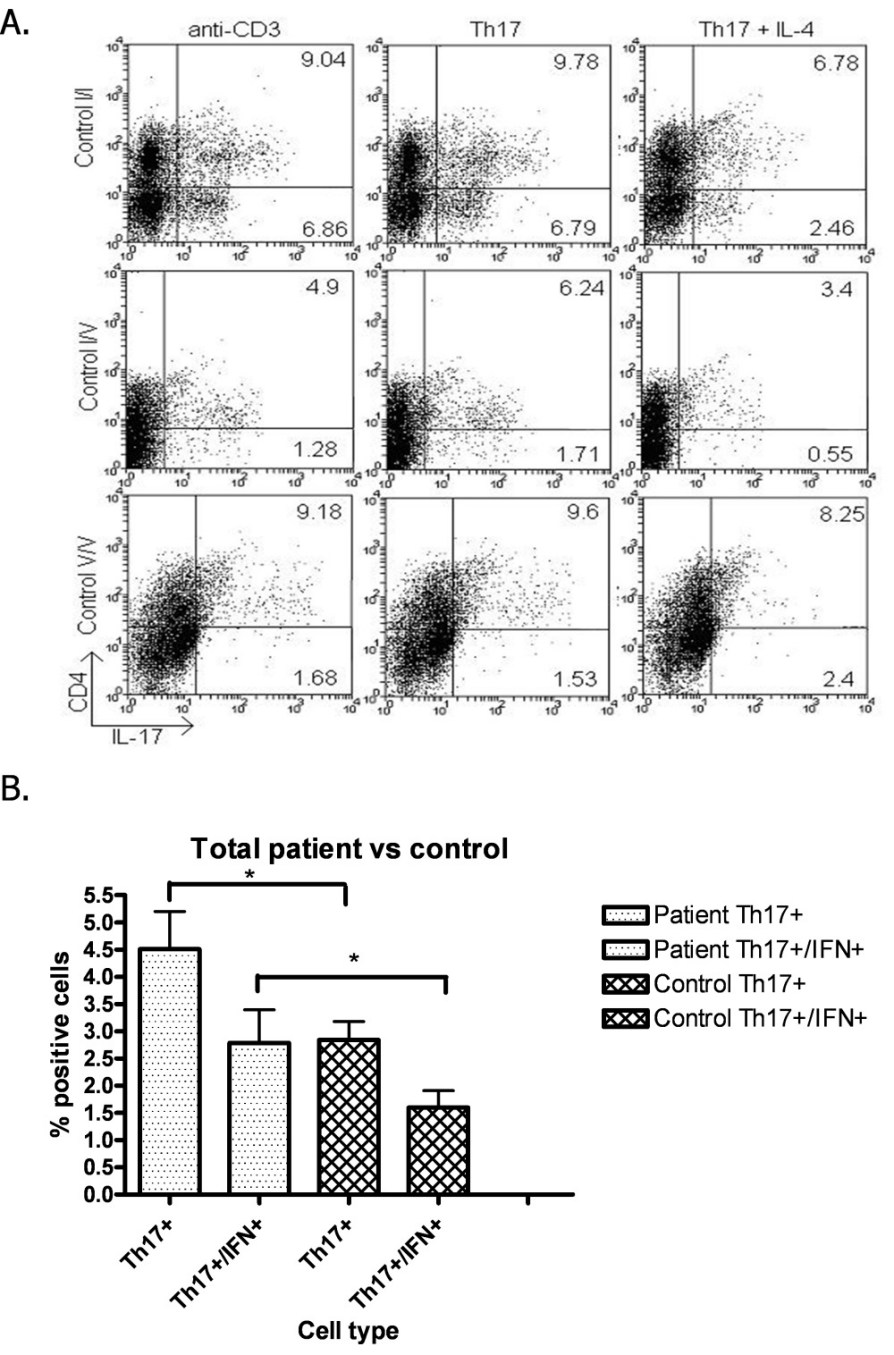
**Flow cytometric enumeration of Th17 cells following a 5-day culture of peripheral blood mononuclear cells (PBMCs)**. **(A) **Representative flow cytometry histograms showing control PBMCs stained for CD4 and interleukin (IL)-17A after stimulation with anti-CD3, anti-CD3 and Th17 stimulatory conditions and anti-CD3 and Th17 stimulatory conditions with IL4. Numbers in quadrants represent the percentage of total cells expressing IL-17A. **(B) **Th17 and Th17/Th1 cell numbers generated in RA patient and control cultures. The difference between each cell type was statistically significant, *P *< 0.05, comparing the patient and control groups. I, isoleucine; V, valine.

We also performed intracellular staining of cultured cells for both IL-17 and IFN-γ and examined the samples by flow cytometry. A set of representative flow cytometry histograms is shown in Figure 3 for each of the healthy control group genotypes. There was a more pronounced suppression of the percentage of IL-17^+ ^cells in the I/I genotype culture, as shown in the top row of Figure 3A, compared to the suppression of IL-17^+ ^cells in the V/V genotype culture, shown in the bottom row. In these cultures, the majority of IL-17^+ ^cells were CD4^+^, but some CD4-IL-17^+ ^cells were also observed. IL-4 likewise affected the expression of IL-17 by these CD4^- ^cells. Flow cytometry of cultured PBMCs activated under Th17 conditions showed that RA patients generated a higher percentage of IL-17^+ ^and IL-17^+^/IFNγ^+ ^(Th1/Th17) cells compared to controls (Figure 3B). A large proportion of the Th17 cells in both healthy individuals and patients with RA are of dual Th17/Th1 lineage. IL-4 generally reduced the number of IL-17^+^/IFNγ^+ ^cells in parallel with reductions in the number of IL-17^+^/IFNγ^- ^cells (data not shown).

### IL-17 concentrations in serum

**Figure 4:**
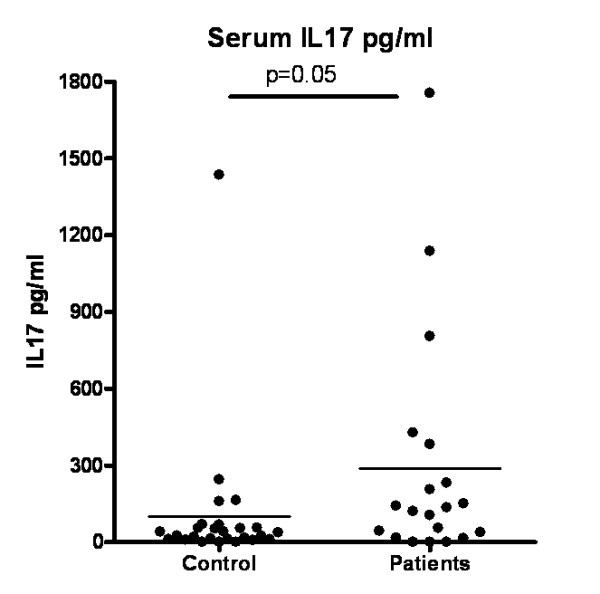
***In vivo *interleukin (IL)-17 production in healthy individuals and RA patients**. Comparison of control and RA serum IL-17 levels, *P *= 0.05.

Consistent with *in vitro *generation of higher numbers of Th17 cells from RA mononuclear cells, we also observed higher serum IL-17 levels in the RA patients compared to the healthy individuals (*P *= 0.05) (Figure 4). These results, as well as the flow cytometry data summarized in Figure 3, are consistent with a recent report that documents expansion of the Th17 subset in RA patients compared to healthy individuals [24].

## Discussion

Several earlier studies supported a key role for IL-17 in the pathogenesis of RA [25]. Determining the regulatory mechanisms that could suppress Th17 cells might lead to novel approaches to the treatment of RA. In this study, we have examined the role of a single nucleotide polymorphism in the *IL-4R *in the control of IL-17 production.

The results indicate that a polymorphism in *IL-4R *in part controls production of IL-17 by Th17 cells cultured from healthy individuals. Specifically, IL-4 significantly inhibited IL-17 production by cells from subjects with the I/I genotype (*P *= 0.0079) and the I/V genotype (*P *= 0.013), but not the V/V genotype. An earlier study showed an association between two copies of the V50 allele and the rapid development of radiographic erosive disease [21]. That report also identified functional effects of the *IL-4R *polymorphism pertinent to Th1 and Th2 cells. With the recent accumulation of information regarding Th17 cells and RA [25], demonstration of a functional impact of the *IL-4R *polymorphism on IL-17 secretion provides further mechanistic insight that could be pertinent to the genetic control of RA severity.

There were several limitations to our current study. The healthy control and RA groups were not precisely matched by age or sex. The *in vitro *data derived from the RA patient group is subject to selection bias due to referral of refractory RA patients to a tertiary center, and this is reflected in the greater prevalence of the V50 allele in this RA sample compared to previous results [21]. The clinical measurements in our patients provide a trend consistent with a previous report that the *IL-4R *is an important severity gene in RA [21]. However, the small sample size precludes any robust claims and points to the need for additional large longitudinal studies of cohorts of patients with early RA.

One study has failed to confirm an association of the I50V polymorphism with RA severity [26]. However, this was a cross-sectional study in which participants had radiographs performed after various durations of RA. Severity was not calculated on the basis of the rate of accumulation of joint damage over a specific interval of time, and therefore an effect of I50V on severity may have been overlooked.

The pattern of IL-17 suppression seen in the healthy individuals was not replicated in the RA patients, potentially because of confounding effects of the various medications. A particularly interesting alternative (but not mutually exclusive) explanation is that in established RA Th17 cells become relatively refractory to IL-4, as we have observed in established CIA [16]. To better assess this possibility, it will be necessary to perform longitudinal studies of *IL-4R *genotype and IL-4-mediated regulation of IL-17 in a cohort of early-onset RA patients.

Allelic variation may lead to either gain or loss of function through the IL-4R. Several prior studies have found that receptors containing isoleucine at position 50, compared with receptors containing valine at the same position, support increased signaling as measured by signal transducer and transactivator 6 phosphorylation [21,27-29]. The precise mechanism for this effect is not yet understood.

Although there is growing evidence for the importance of IL-4 in regulation of IL-17 production, the role that IL-4 plays in controlling inflammation and bone destruction extends beyond regulation of Th17 cells. IL-4 is antiangiogenic [30], and intra-articular injections of the *IL-4 *gene reduced synovial tissue vessel density, inflammation and bone destruction in rat and mouse models of arthritis [31,32]. IL-4 directly suppresses production of vascular endothelial growth factor by synovial fibroblasts [33]. It is not excluded, however, that some of the *in vivo *effects of IL-4 on synovial angiogenesis are due to inhibition of IL-17 production in the synovium, with consequent downregulation of local production of proangiogenic mediators.

Other studies have pointed to a direct role for IL-4 in regulation of tissue destruction in arthritis. IL-4 inhibits the spontaneous and stimulated production of matrix metalloproteinase 1 by synoviocytes [34]. While IL-17 is pro-osteoclastogenic in arthritis [35-37], IL-4 and IL-13 inhibit osteoclastic differentiation by activation of receptors that decrease RANK formation and by activation of receptors on osteoblasts that decrease RANKL expression but increase osteoprotegerin formation [36,38]. In an animal model of osteoarthritis, intra-articular injection of IL-4 inhibits chondrocyte production of nitric oxide and subsequent cartilage destruction [39]. IL-4 may also have suppressive effects on macrophage proliferation [40] and cytokine production [41].

## Conclusions

The data in the present study suggest that a SNP in *IL-4R *confers a hypofunctional receptor that results in decreased inhibition of IL-17 by IL-4, which may allow unrestricted IL-17-mediated inflammation. IL-4 modulates inflammation and joint damage through various mechanisms, including those discussed here, and an attractive topic for future investigation is the effect of this SNP on the ability of IL-4 to regulate pathogenic behavior of cells other than CD4^+ ^Th17 lymphocytes. Genotyping for V50 substitutions in the IL-4R may help identify those patients who are at the greatest risk for inflammation and tissue destruction in RA and who would therefore be the most suitable candidates for aggressive therapy, but this hypothesis requires validation in a prospective study of early RA patients. Approaches that regulate Th17 cells or neutralize their products are under evaluation in the treatment of RA and may be particularly attractive for patients in whom endogenous mechanisms for control of Th17 cells are demonstrably inadequate.

## Abbreviations

APC: antigen-presenting cell; CIA: collagen-induced arthritis; DMARDS: disease-modifying antirheumatic drugs; IFN-γ: interferon-γ; IL: interleukin; MMP: matrix metalloproteinase; NCS: newborn calf serum; PBMC: peripheral blood mononuclear cells; PBS: phosphate-buffered saline; PCR: polymerase chain reaction; PMA: phorbol myristate acetate; RA: rheumatoid arthritis; RANKL: receptor activator of NF-κB ligand; SNP: single-nucleotide polymorphism; STAT: signal transducer and transactivator; TNF: tumor necrosis factor.

## Competing interests

The authors declare that they have no competing interests.

## Authors' contributions

SW participated in study design, performed most of the experiments and drafted the manuscript. LC contributed to study design, optimization of methods, data interpretation and revision of the manuscript. JE supervised implementation of methods and data collection. MJL performed ELISA assays and flow cytometry. JR performed ELISA assays and flow cytometry. ES contributed to study design and performed statistical analysis. DF directed the study design and interpretation of the data and edited the manuscript.

## Supplementary Material

Additional file 1 **Table S1, Supplemental Figures S1 and S2**. Table S1. Baseline characteristics of study patients. Figure S1. Regulation of interleukin-17 production *in vitro*. Figure S2. Inhibition of interleukin (IL)-17 production by IL-4: effect of IL-4R genotype in rheumatoid arthritis patients. Click here for file
