# Supplementary material for: A polymorphism in the interleukin-4 receptor affects the ability of interleukin-4 to regulate Th17 cells: a possible immunoregulatory mechanism for genetic control of the severity of rheumatoid arthritis
Source: Arthritis Res Ther. 2011 Feb 4;13(1):R15. doi: 10.1186/ar3239 (PMC3241359; doi:10.1186/ar3239)
Supplement: Additional file 1 — Table S1, Supplemental Figures S1 and S2. Table S1. Baseline characteristics of study patients. Figure S1. Regulation of interleukin-17 production in vitro. Figure S2. Inhibition of interleukin (IL)-17 production by IL-4: effect of IL-4R genotype in rheumatoid arthritis patients. [file ar3239-S1.DOC]

**Additional Material**

**Supplemental Figure S1. Regulation of IL-17 production *in vitro.***

IL-17A levels (pg/ml) measured by ELISA from supernatants taken from three different culture conditions in RA patients. Calculated p-values are from two-tailed t-tests between IL-17 levels measured by ELISA from cultures containing anti-CD3, anti-CD3 plus Th17 stimulatory conditions and anti-CD3 plus Th17 stimulatory conditions with the addition of IL-4.

**Supplemental Figure S2. Inhibition of IL-17 production by IL-4: effect of IL-4R genotype in RA patients.**

Proportion of IL-17 inhibition by IL-4. Assuming 100% to be the maximal IL-17 production (measured by ELISA) in supernatants of cultures containing anti-CD3 + Th17 stimulatory conditions, the panel represents the percentage change from that baseline after the addition of IL-4 to cell cultures of RA PBMCS.
